# Capsaicin Prevents Contrast-Associated Acute Kidney Injury through Activation of Nrf2 in Mice

**DOI:** 10.1155/2022/1763922

**Published:** 2022-05-16

**Authors:** Fei Ran, Yi Yang, Lun Yang, Shichao Chen, Ping He, Qiting Liu, Qingliang Zou, Dan Wang, Jixin Hou, Peijian Wang

**Affiliations:** ^1^Department of Cardiology, The First Affiliated Hospital, Chengdu Medical College, Chengdu, Sichuan 610500, China; ^2^Sichuan Clinical Research Center for Geriatrics, The First Affiliated Hospital, Chengdu Medical College, Chengdu, Sichuan 610500, China; ^3^Key Laboratory of Aging and Vascular Homeostasis of Sichuan Higher Education Institutes, Chengdu, Sichuan 610500, China

## Abstract

Capsaicin, a transient receptor potential vanilloid 1 channel agonist, possesses antioxidative properties through activating nuclear factor-erythroid 2-related factor 2 (Nrf2). As oxidative stress is a major contributor to the development of contrast-associated acute kidney injury (CA-AKI), we investigated the protective effect of capsaicin against CA-AKI via Nrf2. C57BL/6J mice were treated with dehydration and iodixanol to establish the model of CA-AKI. For pretreatment, capsaicin (0.3 mg/kg) was given via intraperitoneal injection one hour before iodixanol injection. Nrf2-specific siRNA was given through the tail vein to knock down Nrf2. The CA-AKI mouse model had remarkable mitochondrial fragmentation and dysfunction and apoptosis of tubular cells, overproduction of superoxide in renal tubules, increased renal malondialdehyde, tubular epithelial cell injury, and renal dysfunction. Importantly, pretreatment with capsaicin significantly ameliorated tubular cell injury and renal dysfunction with decreased superoxide, renal malondialdehyde, and apoptotic tubular cells and improved mitochondrial morphology and function in the CA-AKI mouse model. The expression of Nrf2 was increased in the kidney from the CA-AKI mouse model and was further enhanced by capsaicin. Administration of siRNA through the tail vein successfully decreased Nrf2 expression in the kidney, and knockdown of Nrf2 by siRNA abolished the beneficial effects of capsaicin on CA-AKI. The present study demonstrated a protective effect of capsaicin pretreatment against CA-AKI via Nrf2.

## 1. Introduction

Acute kidney injury (AKI) is an abrupt loss of renal function, which is strongly associated with increased risk of multiple organ failure and the development of chronic kidney disease. Iodinated contrast media which is widely used in interventional cardiovascular procedures can cause contrast–induced nephropathy, a common cause of AKI. In the clinical practice of nephrology, contrast–induced nephropathy is currently named as contrast-associated acute kidney injury (CA-AKI) as it is challenging to rule out other causes of AKI in clinical settings. The incidence of CA-AKI varies in a wide range from 0% to 24% depending on patient demographic characteristics and comorbidities [1, 2]. To prevent CA-AKI, hydration, intravenous bicarbonate, and pharmacologic prophylaxis with N-acetylcysteine have been used during the periprocedural period. Since the effectiveness and efficacy of these treatments are controversial [3], bicarbonate and N-acetylcysteine are no longer recommended to prevent CA-AKI [4]. Therefore, novel and effective strategies for the prevention of CA-AKI are urgently needed.

Although the mechanisms of CA-AKI are not fully elucidated, mitochondrial oxidative stress and mitochondrial dysfunction are believed to play a crucial role in the development of CA-AKI [5]. Overproduction of mitochondrial reactive oxygen species (ROS) has been proposed as the major culprit that induces apoptosis of renal epithelial cells and subsequently results in acute tubular injury. In addition, excessive production of ROS quenches nitric oxide, leading to arteriolar vasoconstriction and renal ischemic injury. Mitochondrial ROS level is maintained in balance by various oxidases and reductases. Nuclear factor-erythroid 2-related factor 2 (Nrf2) is the master regulator of the cellular and mitochondrial redox homeostasis. As a transcription factor, Nrf2 activation induces transcription and expression of downstream target antioxidant enzymes, which counterbalance the mitochondrial production of ROS [6]. Moreover, activation of Nrf2 improves mitochondrial integrity and function through defending against mitochondrial toxins and promoting mitophagy [7]. A previous study showed that knockout of Nrf2 worsened CA-AKI in mice, suggesting that activation of Nrf2 may attenuate CA-AKI [8]. It has been reported that capsaicin, a transient receptor potential vanilloid 1 (TRPV1) agonist, can activate Nrf2 and induce the expression of the Nrf2 target gene, heme oxygenase-1 (HO-1), in renal tubular cells [9]. Moreover, capsaicin showed renoprotective effects in a variety of animal models [10–12]. Therefore, we hypothesized that activation of Nrf2 with capsaicin could alleviate CA-AKI in an experimental mouse model.

In the present study, capsaicin was given to a mouse model of CA-AKI. In order to evaluate the role of Nrf2, we applied Nrf2-targeting siRNA to the mouse model.

## 2. Materials and Methods

### 2.1. Animals

Male C57BL/6J mice (8-12 weeks of age) were purchased from the Dashuo Biotech Inc. (Chengdu, Sichuan, China) and housed in the animal facility of Chengdu Medical College. The mice were maintained on a 12 h/12 h light/dark cycle with free access to regular chow diet and water. All mouse experiments and protocols were approved by the Institutional Animal Care and Use Committee of Chengdu Medical College, the First Affiliated Hospital. The mice were divided into four groups. The mice in the CA-AKI group were deprived of water for 18 hours and given furosemide (15 mg/kg, i.p.), and 6 hours later, the mice were given iodixanol (15 mg/kg, i.p., GE Healthcare, Shanghai, China) as previously described [13, 14]. The mice in the CA-AKI+CAP group were treated as those in the CA-AKI group plus injection of capsaicin (0.3 mg/kg, i.p.) one hour before iodixanol injection, while capsaicin was replaced with the same volume of saline (0.10-0.16 ml) for mice in the CA-AKI+vehicle group. The mice in the control group were not treated with either water deprivation, furosemide, iodixanol, or capsaicin. Twenty-four hours after iodixanol injection, the mice were euthanized, and the samples were harvested for further analyses.

### 2.2. Knockdown of Nrf2 in Mice

For additional experiments, Nrf2 in mice was knocked down using siRNA as previously described by our group and others [15, 16]. Nrf2-specific siRNA or scrambled control siRNA (50 *μ*g/kg, GenePharma, Shanghai, China) was diluted in 100 *μ*l in vivo-jetPEI (Polyplus, New York, NY, US) and 10% glucose mixture for preparation. The prepared siRNA was administered by hydrodynamic tail vein injection three days prior to iodixanol administration. The sequence of Nrf2-specific siRNA is as follows: sense 5′-UUG GGA UUC ACG CAU AGG AGC ACU G-3′; antisense 5′-CAG UGC UCC UAU GCG GAA UCC CAA-3′.

### 2.3. Assessment of Renal Function

At the end of the experiment, mouse blood was collected by cardiac puncture, and the serum was separated by centrifugation. Blood urea nitrogen (BUN) and creatinine were measured using colorimetric assay kits (Elabscience, Wuhan, China) according to the manufacturer's protocol.

### 2.4. Histology and Immunohistochemistry

The mouse kidneys were harvested 24 hours after iodixanol injection, fixed in 4% paraformaldehyde, embedded in paraffin, and sliced into 6 *μ*m thick sections. The sections were prepared and stained with hematoxylin and eosin. Images were captured using a light microscope (TE2000, Nikon, Toyoko, Japan) and analyzed to evaluate tubular injury. Tubular injury was defined as tubular dilation or atrophy, tubular cell vacuolation, and tubular cell sloughing. Tubular cell injury scores were calculated based on the previously reported scoring system [17]: Score 0: no tubules injured; Score 1: less than 10% of tubules injured; Score 2: 10–24% of tubules injured; Score 3: 25–49% of tubules injured; Score 4: 50–74% of tubules injured; and Score 5: more than 75% of tubules injured. Apoptotic tubular cells in kidney sections were detected using the terminal deoxynucleotidyl transferase dUTP nick end labeling (TUNEL) assay kit (Roche Applied Science, Basel, Switzerland) according to the manufacturer's protocol. The percentage of apoptotic tubular cells was calculated.

### 2.5. Superoxide Assay

Superoxide in renal tubular cells was determined using the dihydroethidium (DHE; Beyotime Biotechnology, Shanghai, China) fluorescent dye [18]. Fresh kidney sections were incubated with DHE (40 *μ*M) for 45 min in a Krebs-Ringer buffer in a dark room. After incubation, the sections were rinsed three times with the Krebs-Ringer buffer. Images were acquired using a microscope (TE2000, Nikon, Toyoko, Japan), and the fluorescence intensity was quantified using the ImageJ software (NIH, USA).

### 2.6. Renal Malondialdehyde (MDA) Assay

Kidney tissue samples were homogenized in ice-cold buffer, and the supernatant was separated and collected. The MDA level was measured using a commercially available kit according to the manufacturer's instruction (#A001-1, Nanjing Jiancheng Bioengineering Institute, Nanjing, Jiangsu, China) [19].

### 2.7. Transmission Electron Microscopy

Mouse kidney tissues were fixed in 4% glutaraldehyde fixative (G1102, Servicebio, Wuhan, China) overnight at 4°C, postfixed, dehydrated, embedded in resin, polymerized at 65°C for 48 h, and sliced into 80 nm thick sections on the ultramicrotome. The sections were mounted on 150-mesh cuprum grids with formvar film. Then, the sections were stained with 2% uranium acetate for 8 min, rinsed in 70% ethanol and ultrapure water for three times, stained in 2.6% lead citrate for 8 min, and then rinsed with ultrapure water. Mitochondria in tubular cells were viewed with a transmission electron microscope (Hitachi HT7800, Hitachi, Tokyo, Japan), and the images were taken with a digital camera (OLYMPUS, Tokyo, Japan) [18].

### 2.8. Assessment of Mitochondrial Membrane Potential

Mitochondria were isolated from fresh kidney cortex tissue as previously described [20], and the isolated mitochondria were then suspended in a mitochondrial storage buffer. Tetraethylbenzimidazolylcarbocyanine iodide (JC-1, Invitrogen, Carlsbad, CA, USA), a cationic carbocyanine dye that highly accumulates in mitochondria, was used to assess mitochondrial membrane potential (*ΔΨ*m). The red and green fluorescence at an excitation wavelength of 490 nm was detected, and the ratio of red/green fluorescence intensity indicated the mitochondrial membrane potential.

### 2.9. Measurement of Adenosine Triphosphate (ATP) Content

The ATP content in the kidney tissue was measured using an ATP assay kit (Cambrex Bio Science, Walkersville, MD, USA) according to the manufacturer's instructions [21]. Briefly, kidney tissue samples were homogenized, and supernatants were collected and used for ATP content measurement. The ATP levels were normalized to total protein content.

### 2.10. Quantitative Real-Time Polymerase Chain Reaction (RT-PCR)

A transverse section of the mouse kidney was snap frozen in liquid nitrogen, and total RNA was isolated with TRIzol (Invitrogen, Carlsbad, CA). Concentration and quality of the extracted RNA were determined using a NanoDrop spectrophotometer (ND 1000, Thermo Fisher Scientific, Waltham, MA, USA). A total 1 *μ*g of RNA was reverse-transcribed into cDNA [22]. The Kim-1 mRNA was quantified by RT-PCR using a SYBR Green Master Mix (Takara, Dalian, Japan). Primer sequences for Kim-1 were as follows: F: 5′-CCT TGT GAG CAC CGT GGC TA-3′; R: 5′-TGT TGT CTT CAG CTC GGG AAT G-3′. The Kim-1 mRNA expression level was normalized to glyceraldehyde 3-phosphate dehydrogenase (GAPDH) mRNA.

### 2.11. Western Blot Analysis

The kidney tissue samples were homogenized, and the total protein was extracted with a radioimmunoprecipitation assay (RIPA) buffer while the nuclear protein was extracted using a Nuclear Extraction Kit (Abcam, Cambridge, MA, USA). The protein concentration was determined using the Protein Assay Kit (Bio-Rad, Hercules, CA, USA). Equal amounts of protein (50 *μ*g) were separated in 10% sodium dodecyl sulfate-polyacrylamide gel electrophoresis and transferred to polyvinylidene fluoride membranes (Bio-Rad) [21]. Membranes were incubated with primary antibodies against caspase-3 (Abcam, 1 : 1000), Bax (Abcam, 1 : 1000), Bcl-2 (Abcam, 1 : 1000), Nrf2 (Abcam, 1 : 1000), HO-1 (Abcam, 1 : 1000), NQO1 (reduced nicotinamide adenine dinucleotide phosphate quinone oxidoreductase 1, Abcam, 1 : 1000), *β*-actin (Abcam, 1 : 10000), GAPDH (Abcam, 1 : 10000), and histone H1 (Abcam, 1 : 5000) overnight at 4°C. After incubation with horseradish peroxidase-conjugated secondary antibodies (ZSGB-Bio, Beijing, China) for 2 h at room temperature, the bands were detected with enhanced chemiluminescence and quantified using a Gel Doc 2000 Imager (Bio-Rad, Hercules, CA, USA). Protein levels of caspase-3, Bax, Bcl-2, HO-1, and NQO1 were normalized to *β*-actin or GAPDH, while the Nrf2 level in the nuclear fraction was normalized to histone H1.

### 2.12. Statistical Analysis

All data are presented as means ± SEM. The differences in mean values among groups were compared using one-way ANOVA with post hoc Tukey honestly significant difference test. A value of *p* < 0.05 was considered statistically significant. The graphs were plotted using GraphPad Prism software version 6.0 (GraphPad Software, La Jolla, CA).

## 3. Results

### 3.1. Capsaicin Prevents Acute Renal Injury and Dysfunction in CA-AKI

**Figure 1:**
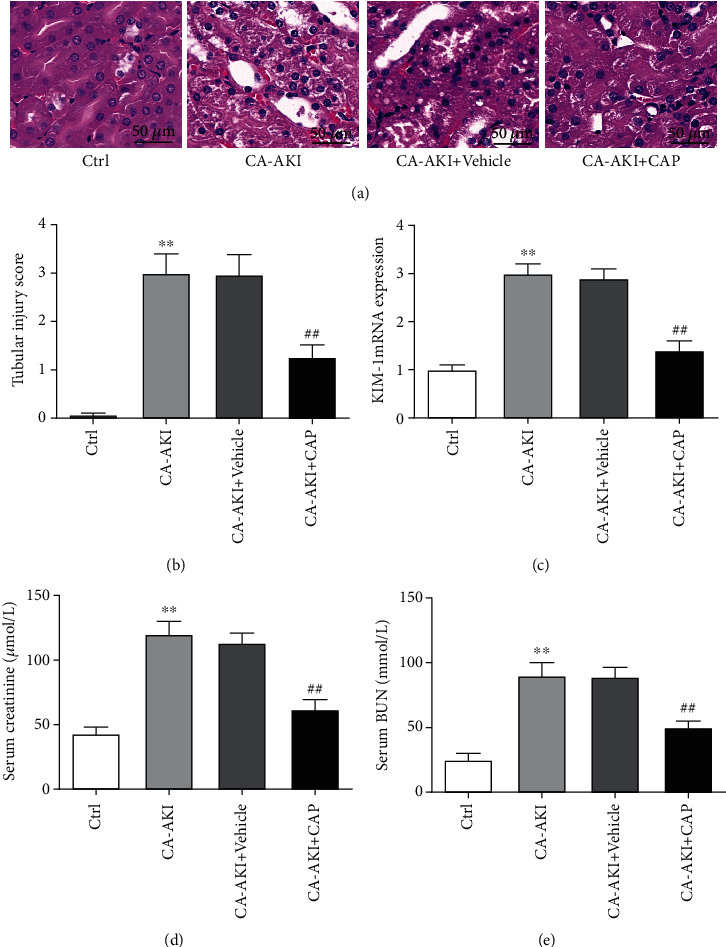
Capsaicin prevents acute renal injury and dysfunction in CA-AKI. (a) Representative hematoxylin and eosin-stained sections of the kidney from mice in the control, CA-AKI, CA-AKI+vehicle, and CA-AKI+CAP groups. Quantification of tubular injury score (b), mRNA expression levels of Kim-1 (c), serum creatinine (d), and BUN (e) of mice in the control, CA-AKI, CA-AKI+vehicle, and CA-AKI+CAP groups. Data are means ± SEM. *N* = 8 in each group. ^∗∗^*p* < 0.01 vs. Ctrl; ^##^*p* < 0.01 vs. CA-AKI.

Renal tubular cells in the kidney sections from mice in the CA-AKI group displayed vacuolation degeneration, necrosis, and sloughing with remarkably increased tubular injury scores when compared with those from control mice (Figures 1(a) and 1(b), *p* < 0.01). Pretreatment with capsaicin significantly attenuated tubular cell degeneration and tubular injury scores in CA-AKI (Figures 1(a) and 1(b), *p* < 0.01). The mRNA expression of Kim-1, a biomarker upregulated in proximal tubular cells following kidney injury, was abruptly increased in the renal tissue from mice in the CA-AKI group compared with control mice (Figure 1(c), *p* < 0.01), while pretreatment with capsaicin blunted the upregulation of KIM-1 in CA-AKI (Figure 1(c), *p* < 0.01). Mice in the CA-AKI group had remarked renal dysfunction reflected as elevated levels of serum creatinine (Figure 1(d), *p* < 0.01) and BUN (Figure 1(e), *p* < 0.01), which were significantly attenuated in CA-AKI mice pretreated with capsaicin (Figures 1(d) and 1(e), both *p* < 0.01).

### 3.2. Capsaicin Prevents Renal Tubular Cell Apoptosis in CA-AKI

**Figure 2:**
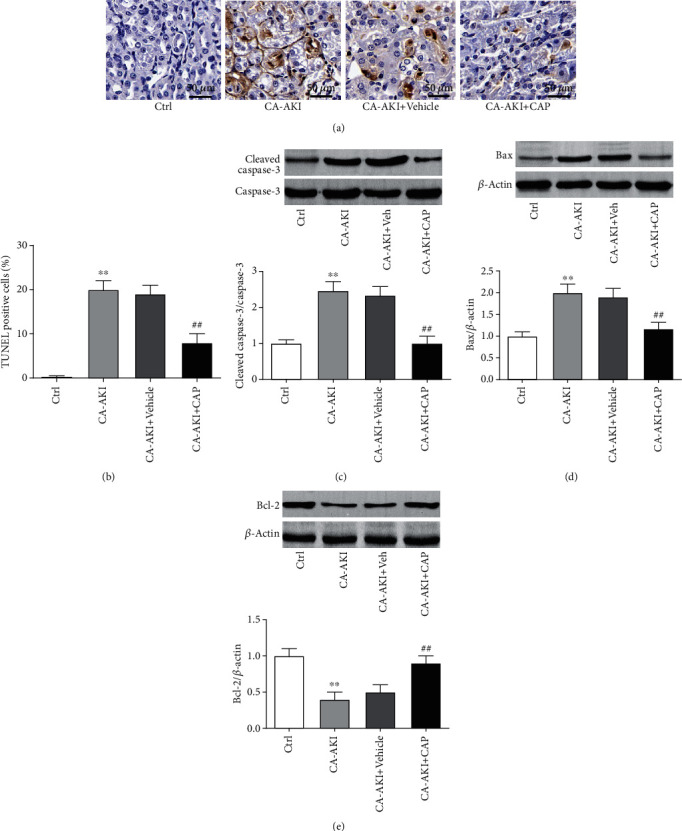
Capsaicin prevents renal tubular cell apoptosis in CA-AKI. (a) Representative immunohistochemistry staining of TUNEL in sections of the kidney from mice in the control, CA-AKI, CA-AKI+vehicle, and CA-AKI+CAP groups. Quantification of TUNEL-positive cells (b) and representative Western blotting bands and protein expression quantification of cleaved caspase-3 (c), Bax (d), and Bcl-2 (e) in the kidney of mice in the control, CA-AKI, CA-AKI+vehicle, and CA-AKI+CAP groups. Data are means ± SEM. *N* = 8 in each group. ^∗∗^*p* < 0.01 vs. Ctrl; ^##^*p* < 0.01 vs. CA-AKI.

As shown in Figure 2(a) and quantified in Figure 2(b), the kidney sections from the mice in the CA-AKI group had more TUNEL-positive apoptotic renal tubular cells than those from control mice, while TUNEL-positive apoptotic cells in the kidney sections from CA-AKI mice with capsaicin pretreatment were less than those from either iodixanol- or vehicle-treated mice. To further study the antiapoptotic effects of capsaicin, we measured the expression levels of apoptosis-associated proteins in renal tissue by Western blotting. The level of cleaved caspase-3 was significantly increased in the CA-AKI group compared with the control mice (Figure 2(c), *p* < 0.01) and so was the expression of the proapoptotic protein Bax (Figure 2(d), *p* < 0.01). In contrast, the expression of the antiapoptotic protein Bcl-2 was remarkably decreased in the CA-AKI group compared with the control group (Figure 2(e), *p* < 0.01). Importantly, the increases in cleaved caspase-3 and Bax and the decrease in Bcl-2 were almost abolished by pretreatment of capsaicin (all *p* < 0.01) but not by the vehicle (Figures 2(c)–2(e)).

### 3.3. Capsaicin Prevents Oxidative Stress in Tubular Cells in CA-AKI

**Figure 3:**
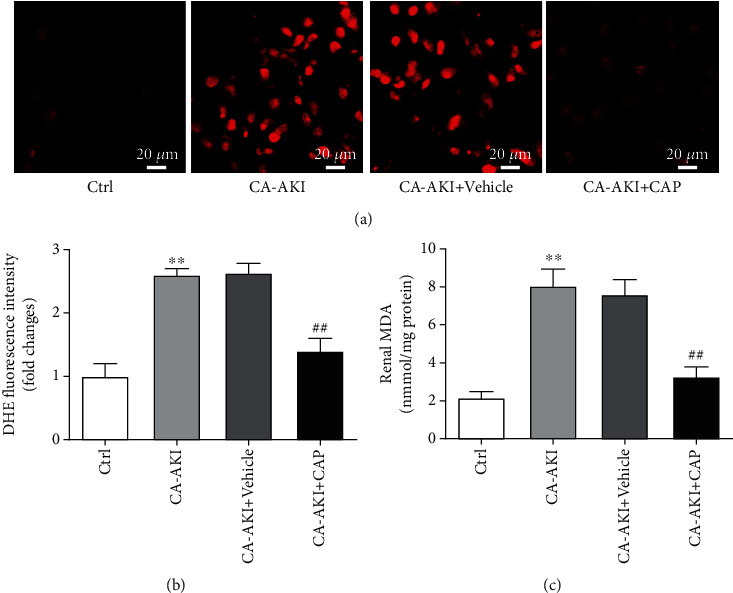
Capsaicin prevents oxidative stress in tubular cells in CA-AKI. (a) Representative DHE fluorescence staining of fresh renal sections from mice in the control, CA-AKI, CA-AKI+vehicle, and CA-AKI+CAP groups. (b) Quantification of DHE fluorescence intensity. (c) Renal malondialdehyde (MDA) levels of mice in the control, CA-AKI, CA-AKI+vehicle, and CA-AKI+CAP groups. Data are means ± SEM. *N* = 8 in each group. ^∗∗^*p* < 0.01 vs. Ctrl; ^##^*p* < 0.01 vs. CA-AKI.

As oxidative stress is an important mechanism of iodixanol-induced cell apoptosis, we detected superoxide, a major component of reactive oxygen species, in the kidney using the DHE fluorescent dye. We viewed the images of the renal tubule fields and quantified the DHE intensity in tubular cells. The results showed that mice in CA-AKI had significantly increased superoxide levels in tubular cells (Figures 3(a) and 3(b), *p* < 0.01), while pretreatment with capsaicin attenuated overproduction of superoxide in CA-AKI (Figures 3(a) and 3(b), *p* < 0.01). Similar results were observed in the measurement of renal tissue MDA levels (Figure 3(c)).

### 3.4. Capsaicin Ameliorates Mitochondrial Dysfunction of Tubular Cells in CA-AKI

**Figure 4:**
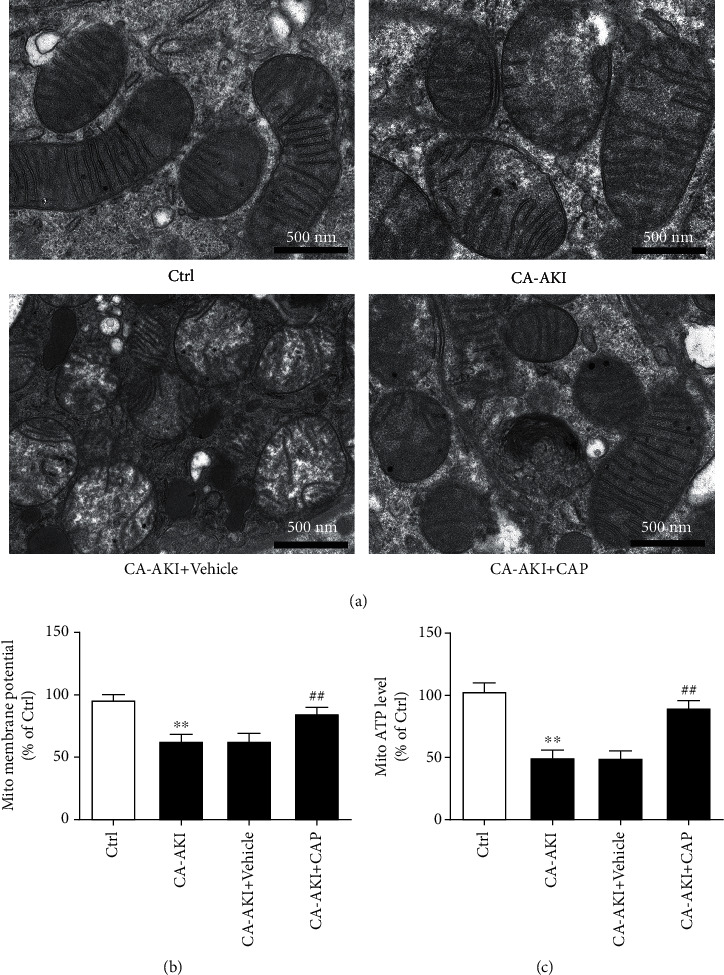
Capsaicin ameliorates mitochondrial dysfunction of tubular cells in CA-AKI. (a) Representative transmission electron microscopic images of mitochondria in the tubular cells from mice in the control, CA-AKI, CA-AKI+vehicle, and CA-AKI+CAP groups. Mitochondrial membrane potential (b) and mitochondrial ATP level (c) of mice in the control, CA-AKI, CA-AKI+vehicle, and CA-AKI+CAP groups. Data are means ± SEM. *N* = 8 in each group. ^∗∗^*p* < 0.01 vs. Ctrl; ^##^*p* < 0.01 vs. CA-AKI.

Mitochondria are the most important organelles that are involved in cell injury and apoptosis. Mitochondria in tubular cells were observed under electron microscopy. In mice from the CA-AKI group, mitochondria were defectively enlarged with disarranged and ruptured cristae (Figure 4(a)), which were obviously improved in those from the mice pretreated with capsaicin (Figure 4(a)). To quantify the mitochondrial dysfunction, mitochondrial membrane potential and ATP levels in renal tubular cells were determined. Renal tubular cells from mice in the CA-AKI group had remarkably reduced mitochondrial membrane potential and ATP levels (Figures 4(b) and 4(c), *p* < 0.01), and pretreatment with capsaicin nearly reversed CA-AKI-associated decreases in the mitochondrial membrane potential and ATP levels (Figures 4(b) and 4(c), *p* < 0.01).

### 3.5. Knockdown of Nrf2 Abolishes the Renoprotective Effects of Capsaicin in CA-AKI

**Figure 5:**
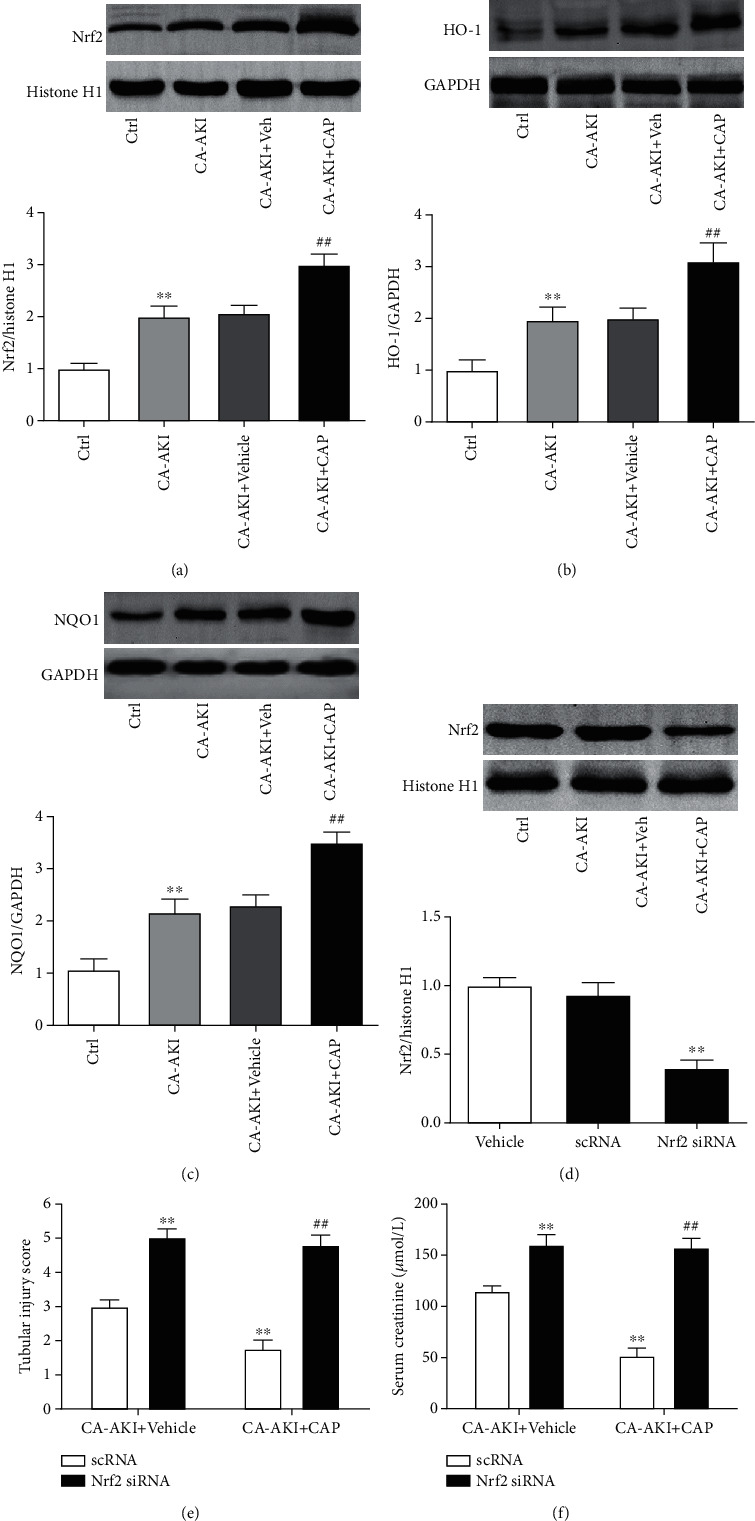
Knockdown of Nrf2 abolishes the renoprotective effects of capsaicin in CA-AKI. Representative Western blotting and protein expression quantification of Nrf2 (a), HO-1 (b), and NQO1 (c) in the kidney from mice in the control, CA-AKI, CA-AKI+vehicle, and CA-AKI+CAP groups. ^∗∗^*p* < 0.01 vs. Ctrl; ^##^*p* < 0.01 vs. CA-AKI. (d) Representative Western blotting and protein expression quantification of Nrf2 in the kidney from mice treated with vehicle, scrambled siRNA (scRNA), and Nrf2-specific siRNA. ^∗∗^*p* < 0.01 vs. vehicle. Tubular injury score (e) and serum creatinine levels (f) of scRNA- or siRNA-injected mice in the CA-AKI or CA-AKI-plus-capsaicin group. Data are means ± SEM. *N* = 8 in each group. ^∗∗^*p* < 0.01 vs. scRNA-treated CA-AKI mice; ^##^*p* < 0.01 vs. scRNA-treated CA-AKI-plus-capsaicin mice.

**Figure 6:**
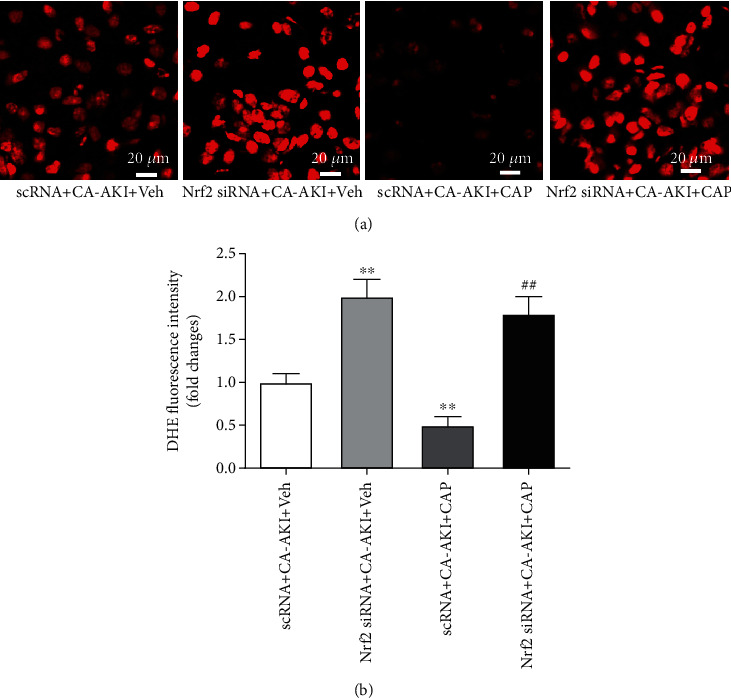
Knockdown of Nrf2 abolishes the antioxidative stress effect of capsaicin in CA-AKI. (a) Representative DHE fluorescence staining of fresh renal sections from scRNA- or siRNA-injected mice in the CA-AKI or CA-AKI-plus-capsaicin group. (b) Quantification of DHE fluorescence intensity. Data are means ± SEM. *N* = 8 in each group. ^∗∗^*p* < 0.01 vs. scRNA-treated CA-AKI mice; ^##^*p* < 0.01 vs. scRNA-treated CA-AKI-plus-capsaicin mice.

**Figure 7:**
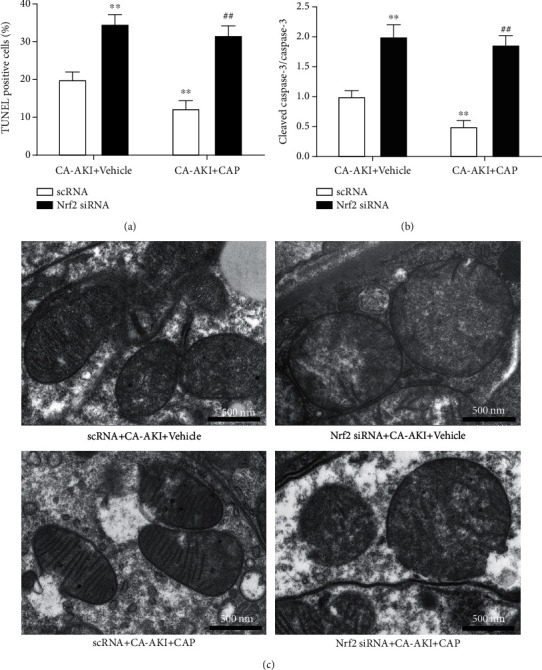
Knockdown of Nrf2 abolishes antiapoptotic effects of capsaicin in CA-AKI. TUNEL-positive tubular cells (a) and cleaved caspase-3 expression level (b) of scRNA- or siRNA-injected mice in the CA-AKI or CA-AKI-plus-capsaicin group. Data are means ± SEM. *N* = 8 in each group. ^∗∗^*p* < 0.01 vs. scRNA-treated CA-AKI mice; ^##^*p* < 0.01 vs. scRNA-treated CA-AKI-plus-capsaicin mice. (c) Representative transmission electron microscopic images of mitochondria in the tubular cells from scRNA- or siRNA-injected mice in the CA-AKI or CA-AKI-plus-capsaicin group.

As Nrf2 mediates capsaicin's antioxidative effects, we investigated the role of Nrf2 in the renoprotective effects of capsaicin. We found that the expression of Nrf2 in the kidney was significantly increased in mice from the CA-AKI group (Figure 5(a), *p* < 0.01) and was further enhanced in the CA-AKI+CAP group (Figure 5(a), *p* < 0.01). Similar changes were observed in the protein levels of HO-1 and NQO1, the target genes of Nrf2 (Figures 5(b) and 5(c)). Next, Nrf2 in the kidney was knocked down by injecting siRNA (Figure 5(d), *p* < 0.01). Mice with or without Nrf2 knockdown were subjected to establishment of the CA-AKI model. Knockdown of Nrf2 exacerbated CA-AKI-associated renal tubular injury and dysfunction, reflected as increased tubular injury score and serum creatinine levels, respectively (Figures 5(e) and 5(f), both *p* < 0.01). Interestingly, pretreatment with capsaicin failed to improve CA-AKI-associated renal tubular injury and dysfunction in Nrf2 knockdown mice (Figures 5(e) and 5(f)). Similarly, Nrf2 knockdown abolished the antioxidative stress effect of capsaicin (Figures 6(a) and 6(b)). Knockdown of Nrf2 worsened CA-AKI-associated tubular cell apoptosis with more TUNEL-positive cells (Figure 7(a), *p* < 0.01) and increased cleaved caspase-3 (Figure 7(b), *p* < 0.01), and Nrf2 knockdown abolished capsaicin's antiapoptotic effects (Figures 7(a) and 7(b)). Moreover, capsaicin failed to improve CA-AKI-associated mitochondrial swelling and fragmentation in tubular cells from Nrf2 knockdown mice (Figure 7(c)).

## 4. Discussion

In this study, we found that capsaicin prevents mitochondrial dysfunction, oxidative stress, apoptosis of renal tubular cells, and renal dysfunction in a mouse model of CA-AKI induced by a combination of dehydration, furosemide, and iodixanol. More importantly, the preventative effects of capsaicin in CA-AKI are mediated by the antioxidant and anti-inflammatory transcription factor Nrf2.

Capsaicin is a chemical compound and an ingredient that was originally isolated from chili peppers. Currently, capsaicin is not only broadly present in many foods from a variety of cultures but also used as a medicine to treat arthritis and musculoskeletal pain based on its anti-inflammatory and analgesic activities. Since the antioxidant and anti-inflammatory properties of capsaicin were noticed, its renoprotective effects have been investigated in various animal models [23, 24]. Ischemia/reperfusion-induced renal injury is a prototype model for the study of AKI. It has been reported that administration of capsaicin or its ultrapotent analog, resiniferatoxin, could prevent ischemia/reperfusion-induced renal injury and dysfunction in rats likely through inhibition of inflammatory response [25, 26]. Experimental studies also found that capsaicin attenuated renal ischemia/reperfusion injury-induced enhancement of salt sensitivity and prevented salt-induced kidney injury and hypertension after acute renal ischemia-reperfusion injury in rats [10, 27]. In addition to ischemia/reperfusion-induced renal injury, capsaicin also provides beneficial effects on other types of AKI. Cisplatin, an antineoplastic medication, is notorious for causing nephrotoxicity when used in the treatment of many solid-organ cancers [28]. Cisplatin-indued nephrotoxicity is largely mediated by inducing tubular cell injury and death and can be exacerbated by administration of contrast media [29, 30]. It has been reported that capsaicin could prevent cisplatin-induced nephrotoxicity and AKI via inhibition of oxidative stress and inflammation [9, 11]. Although CA-AKI shares similar pathogenic mechanisms with other types of AKI, the beneficial effects of capsaicin in CA-AKI have yet to be reported. Therefore, the present study provides a novel renoprotective role of capsaicin in CA-AKI. Besides AKI, chronic administration of capsaicin induced diuresis and lowered renal injury biomarker levels in rats with experimental diabetes [31]. Moreover, a longitudinal large-scale population-based study demonstrated that chili consumption was inversely associated with the progression of chronic kidney disease, suggesting a renoprotective effect of capsaicin in patients [32]. In general, capsaicin holds the potential to be used as a renoprotective agent.

Capsaicin is a natural agonist of TRPV1. TRPV1, also known as the capsaicin receptor and the vanilloid receptor 1, is a nonselective calcium-permeable cation channel. Activation of TRPV1 enables calcium entry and mediates various signaling pathways and cellular processes. Accumulative evidence shows that TRPV1 exerts renoprotective properties upon activation. Knockout of TRPV1 exacerbated ischemia/reperfusion-induced renal inflammation and injury, and ablation of TRPV1-positive renal afferent nerves worsened salt-induced hypertension and renal injury in rats after renal ischemia/reperfusion injury via enhanced inflammation and oxidative stress [33, 34]. In addition, previous studies reported that the renoprotective effects of capsaicin in animal models were dependent on activation of TRPV1 [10, 27]. Furthermore, activation of TRPV1 with agonists other than capsaicin, such as N-octanoyl-dopamine, could also ameliorate AKI [35]. Based on previous studies, TRPV1 channels expressed in both afferent renal nerves and tubular cells could contribute to the renoprotective role of TRPV1 activation. Activation of TRPV1 in afferent renal nerves induces release of sensory neuropeptides such as calcitonin gene-related peptide (CGRP) which has antioxidant and anti-inflammatory properties [36, 37]. A supporting study demonstrated that pretreatment with CGRP alleviated ischemia/reperfusion-induced AKI in rats [38]. Oxidative stress induces apoptosis of renal tubular cells likely through caspase activation [39]. Thus, capsaicin might protect renal tubular cells from apoptosis via suppression of oxidative stress. On the other hand, TRPV1 is also expressed in tubular epithelial cells and exerts diuretic effects when activated by capsaicin [40]. In the present study, Trpv1 gene knockout mice were not used to test whether the beneficial effects of capsaicin on CA-AKI were mediated by TRPV1. It has been noted that capsaicin could regulate reactive oxygen species levels in cells in a TRPV1-independent manner [41]. Therefore, the renoprotective effects of capsaicin could be TRPV1-indepednent or TRPV1-dependent on TRPV1 channels in either renal afferent nerves or renal tubular cells.

Nrf2 is a master regulator of endogenous antioxidant systems by acting as a transcription factor and controls the glutathione and thioredoxin antioxidant systems [42, 43]. The role of Nrf2 in CA-AKI has recently been focused as quite a few agents are reported to prevent CA-AKI via Nrf2 [8, 44–46]. A variety of natural compounds including capsaicin have been considered as Nrf2 activators [47, 48]. After being activated, Nrf2 will be translocated into the nucleus and will then initiate transcription of downstream target genes. It has been reported that capsaicin could upregulate HO-1, one of the Nrf2 target genes [9]. In the present study, the increase of Nrf2 expression in the kidney from mice treated with iodixanol could be a compensatory upregulation against renal injury. We found that capsaicin further increased the expression of Nrf2 in the kidney and that knockdown of Nrf2 almost abolished the beneficial effects of capsaicin in the animal model of CA-AKI. These findings strongly suggest that Nrf2 mediates the effects of capsaicin in CA-AKI.

Although capsaicin is a promising renoprotective agent, it is still challenging to develop a capsaicin-based treatment against CA-AKI. Currently, capsaicin cream has been used topically as an analgesic, whereas capsaicin when given orally may cause burning or irritation. This is a proof-of-concept pilot study. One of the limitations is that Trpv1 and Nrf2 gene knockout mice were not used to elucidate the underlying mechanism for the therapeutic effects of capsaicin. Another limitation of the present study is the lack of *in vitro* experiments to explore the underlying mechanism for the therapeutic effects of capsaicin.

## 5. Conclusions

In conclusion, the present study demonstrated a protective effect of capsaicin against CA-AKI via Nrf2. The present study provides an avenue for using capsaicin as a promising therapeutic agent for the prevention or treatment of CA-AKI.

## Data Availability

The experimental data used to support the findings of this study are available from the corresponding author upon request.

## Abbreviations

AKI: Acute kidney injury
CA-AKI: Contrast-associated acute kidney injury
ROS: Reactive oxygen species
Nrf2: Nuclear factor erythroid-related factor 2
TRPV1: Transient receptor potential vanilloid 1
HO-1: Heme oxygenase-1
BUN: Blood urea nitrogen
TUNEL: Terminal deoxynucleotidyl transferase dUTP nick end labeling
DHE: Dihydroethidium
MDA: Malondialdehyde
ATP: Adenosine triphosphate
RT-PCR: Quantitative real-time polymerase chain reaction
GAPDH: Glyceraldehyde 3-phosphate dehydrogenase
NQO1: Reduced nicotinamide adenine dinucleotide phosphate quinone oxidoreductase 1.
